# Agroforestry trade-offs between biomass provision and aboveground carbon sequestration in the alpine Eisenwurzen region, Austria

**DOI:** 10.1007/s10113-021-01794-y

**Published:** 2021-07-21

**Authors:** Bastian Bertsch-Hoermann, Claudine Egger, Veronika Gaube, Simone Gingrich

**Affiliations:** Institute of Social Ecology (SEC), Department of Economics and Social Sciences (WiSo), University of Natural Resources & Life Sciences, Vienna (BOKU), Schottenfeldgasse 29, 1070 Vienna, Austria

**Keywords:** Mountain agriculture, Multi-functionality, Land use policy, Ecosystem services, Long-term socio-ecological research (LTSER)

## Abstract

Mountain agroecosystems deliver essential ecosystem services to society but are prone to climate change as well as socio-economic pressures, making multi-functional land systems increasingly central to sustainable mountain land use policy. Agroforestry, the combination of woody vegetation with crops and/or livestock, is expected to simultaneously increase provisioning and regulating ecosystem services, but knowledge gaps concerning trade-offs exist especially in temperate industrialized and alpine regions. Here, we quantify the aboveground carbon (C) dynamics of a hypothetical agroforestry implementation in the Austrian long-term socio-ecological research region *Eisenwurzen* from 2020 to 2050. We develop three land use scenarios to differentiate conventional agriculture from an immediate and a gradual agroforestry implementation, integrate data from three distinct models (Yield-SAFE, SECLAND, MIAMI), and advance the socio-ecological indicator framework Human Appropriation of Net Primary Production (HANPP) to assess trade-offs between biomass provision and carbon sequestration. Results indicate that agroforestry strongly decreases HANPP because of a reduction in biomass harvest by up to − 47% and a simultaneous increase in actual net primary production by up to 31%, with a large amount of carbon sequestered in perennial biomass by up to 3.4 t C ha^-1^ yr^-1^. This shows that a hypothetical transition to agroforestry in the *Eisenwurzen* relieves the agroecosystem from human-induced pressure but results in significant trade-offs between biomass provision and carbon sequestration. We thus conclude that while harvest losses inhibit large-scale implementation in intensively used agricultural regions, agroforestry constitutes a valuable addition to sustainable land use policy, in particular when affecting extensive pastures and meadows in alpine landscapes.

**Supplementary Information:**

The online version contains supplementary material available at 10.1007/s10113-021-01794-y.

## Introduction

Land use change is a major driver of global environmental change, degrading ecosystems and contributing to climate change (IPCC 2019; Ellis et al. 2013; Turner et al. 2007; Foley 2005). In order to reconcile projected increases in biomass demand for food and energy (Camia et al. 2018; Coelho et al. 2012; Tilman 2001) while minimizing land demand for agriculture and negative environmental impacts of conventional farming, the concept of ecological intensification is increasingly propagated (e.g., Tittonell 2014; Bommarco et al. 2013). Ecological intensification refers to the design and management of agricultural systems to foster regulating ecosystem services (ES), which minimize environmental degradation and sustain or increase production (Kleijn et al. 2019). How to integrate the maximization of provisioning and regulating ES, however, remains a crucial question to be addressed (Grass et al. 2020).

ES trade-offs arise when the increased use of one ES, e.g., food production, results in the decreased provision of another ES, e.g., carbon sequestration (Rodríguez et al. 2006). ES trade-offs, in particular in mountain ecosystems, are subject to complex interrelationships between environmental, biological, technological, and socio-economic conditions (Briner et al. 2013b). Mountain ecosystems provide critical ES (Egan and Price 2017; Grêt-Regamey et al. 2012) and thus present important case studies to investigate trade-offs between biomass provision and carbon sequestration involved with different agricultural practices. Land use in the European Alps has a major impact on local biogeochemical cycles and ecosystem structure and functions (Tasser et al. 2005). Land use change in alpine and pre-alpine regions, including the Eisenwurzen, is mainly characterized by abandonment of once extensively managed land and intensification on favorable plots (Streifeneder et al. 2007; Flury et al. 2013; Vigl 2016). Land use change is thereby being the most important driver for biodiversity loss (Zimmermann 2010; Tasser and Tappeiner 2002). Additionally, mountain environments experience more rapid climatic change than environments at lower elevations, which in turn accelerates the rate of ecosystem change and increasingly threatens the provision of important ES (Seidl et al. 2019; Schirpke et al. 2017; Mountain Research Initiative EDW Working Group 2015; Gobiet et al. 2014; Dirnböck et al. 2011). The concept of multi-functionality (Manning et al. 2018) is becoming increasingly central in response to the urgent question how to adapt agriculture in the Alps towards climate neutrality and resilience (Lavorel et al. 2017; Flury et al. 2013; Huber et al. 2012), taking into account the cultural and natural heritage as well as the complexity of socio-ecological interactions (Huber 2020; Alpine Convention 2019; Fleury et al. 2008).

Agroforestry, the combination of woody vegetation with crops and/or livestock on the same unit of land, including wood pastures and extensive orchard meadows as well as intensive tree-crop systems (Mosquera-Losada et al. 2018; Nair et al. 2008), has been shown to be a sustainable, multi-functional land system with various potentially positive ecological and socio-economic effects, reconciling climate change mitigation and adaptation (Matocha et al. 2012). In addition to biomass provision, benefits of agroforestry pertain to enhancing biodiversity; regulating soil, water, and air quality; supporting biological pest control; more efficient nutrient cycling; and positively modifying micro- to macro-climates, all together reducing greenhouse gas emissions as well as the need for external inputs (Lawson et al. 2019; Kay et al. 2019; Torralba et al. 2016; Smith et al. 2013; McAdam et al. 2008).

Wood pastures and extensive orchard meadows are historically important agroforestry systems in central Europe and the alpine region, enhancing the ecological and social integrity of agricultural landscapes (Herzog 1998; Hartel et al. 2015). Nevertheless, these land systems have deteriorated for decades due to adverse socio-economic effects such as increased competition from intensively managed orchards, decreased fruit market prices, and insufficient agri-environmental policies (Schönhart et al. 2011; Herzog 1998). In the alpine and subalpine belts, wood pastures mainly occur at an elevation of up to 1400 m a.s.l. and extensive meadow orchards up to 1200 m a.s.l. (Herzog 1998; Buttler et al. 2008). Conservation and re-establishment of these two agroforestry systems are of particular interest considering sustainable land use and ecological restoration planning in mountain areas (Buttler et al. 2008; Bergmeier et al. 2010), providing structural- and species-rich landscapes enhancing ecological connectivity and reducing nitrate leeching, as well as providing a high aesthetic and cultural value relevant for the local population and tourism (Herzog 1998; Helga et al. 2005). In particular, extensive orchard meadows are a traditional and characteristic form of land use in the region and related activities are part of regional development initiatives (e.g., Styrian Eisenwurzen Nature & Geopark 2020) as well as the Austrian Agri-Environmental Programme (ÖPUL) and other federal subsidies (Gantar et al. 2011).

While agroforestry’s carbon sequestration potential has been addressed in numerous studies (Lawson et al. 2019; Feliciano et al. 2018; Aertsens et al. 2013; Ramachandran Nair et al. 2010; Dixon 1995), effects on food production are less clear. Many studies that show agroforestry sustaining or increasing food security focus on subsistence farming in the Global South (Niether et al. 2020; Montagnini and Metzel 2017; Mbow et al. 2014), while evaluation of impacts on the productivity in highly efficient agricultural systems shows mostly negative, but also neutral and positive yield effects (Lehmann et al. 2020; Swieter et al. 2019; Pardon et al. 2018; Arenas-Corraliza et al. 2018; Rivest et al. 2013). Further assessments of ES trade-offs, in particular in temperate and industrialized mountain agroecosystems, will be useful for informing policy decisions (Miller et al. 2020; Brown et al. 2018).

This study contributes to the debate on agroforestry’s potential for ecological intensification in temperate industrialized and alpine regions by quantifying aboveground carbon (C) dynamics of a hypothetical transition to silvoarable agroforestry with wild cherry (*Prunus avium L.*) in the Austrian alpine Eisenwurzen region. To this end, we quantify trade-offs between provisioning ES (biomass harvest) and regulating ES (aboveground carbon sequestration through biomass accumulation) in three land use scenarios. The scenarios serve to differentiate between (i) conventional agriculture, (ii) immediate implementation of agroforestry in the year 2020, and (iii) gradual implementation of agroforestry between 2020 and 2045. Results will thus enable to identify and quantify the trade-offs between carbon sequestration and biomass production under two divergent trajectories of agroforestry implementation, compared to conventional agriculture. Our aim is to shed light on the biophysical potentials of and barriers to agroforestry as a measure for ecological intensification in the context of industrialized and alpine agriculture.

## Materials and methods

### Study region

The study region (Fig. 2) is part of the long-term socio-ecological research platform *Eisenwurzen* in the Austrian Northern Limestone Alps, covering an area of 1425 km^2^ between 250 and 2369 m a.s.l. The topographic gradient ranges from the low-lying Danube basin in the north over hilly and montane grasslands and forests to the alpine peaks of the Gesäuse in the south. It covers a heterogeneous representative transect of topography and land use in Austria and is well suited to approach the question of sustainable mountain agriculture while embedding the issue in a broader socio-ecological context (Gingrich et al. 2016).

In the twentieth century, the region was characterized by typical alpine land use trends of regional specialization (Krausmann et al. 2003; Gingrich et al. 2018). This entails (i) primarily intensive arable and livestock agriculture in the north, where only small-scale forest islands remain; (ii) intensive and, to a lesser degree, extensive grassland agriculture including the last remains of historic orchard meadows in the hilly and submontane landscape, with forestry playing a secondary role; and (iii) mostly intensive grasslands in the valleys as well as increasingly abandoned extensive alpine pastures and meadows in the rugged south, where > 85% are covered with forests (Draschan et al. 2003; Helga et al. 2005; Gingrich et al. 2013).

To represent cropland and grassland, the two major land use types addressed in this study, two modeling sites were chosen in locations dominated by cropland and grassland, respectively (Fig. 2a). Modeling site A (N 48.160073, E 14.451603) is situated in the Danube basin at c. 270 m a.s.l. It is characterized by good soil conditions (mostly unconsolidated brown earth sediment) and a moderate climate (8–9 °C mean annual temperature, 800–1000 mm annual precipitation). Intensively managed cropland (primarily cereals and maize) dominates this region, while grassland is only found on less fertile plots. Modeling site B (N 47.945322, E 14.443828) is located at c. 670 m a.s.l. in the hilly and submontane region. The climate is slightly cooler and more humid (6–9 °C mean annual temperature, 1166–1560 mm annual precipitation) and soils are mostly fine-grained and densely packed brown loam exhibiting only average productivity (Helga et al. 2005). This area is dominated by intensively managed meadows while pastures are only found on steeper terrain. At both sites, intensive agriculture results in structurally poor agroecosystems, biodiversity loss, and groundwater contamination (Draschan et al. 2003; Geissler et al. 2003).

### Methodological framework

**Fig. 1:**
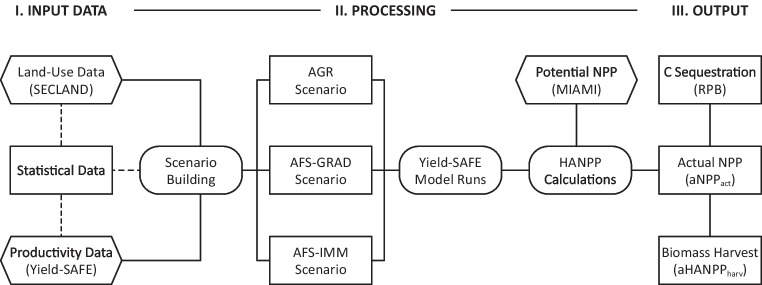
Methodological framework depicting input data, processing steps, and output. Hexagons refer to model data and rounded rectangles to processing steps. Land use scenarios include conventional agriculture (AGR) as well as immediate and gradual implementation of agroforestry (AFS-IMM and AFS-GRAD). Outputs only illustrate the three central indicators in this study, i.e., actual aboveground net primary production (aNPP_act_), remaining perennial biomass (RPB), and aboveground biomass harvest (aHANPP_harv_). A full description of indicators included in the Human Appropriation of Net Primary Production (HANPP) framework is found in the corresponding section below

The methodological framework combines data from three distinct modeling approaches and regional-level agricultural statistics to formulate one agriculture and two agroforestry scenarios and quantify inherent carbon dynamics with an extended version of the Human Appropriation of Net Primary Production (HANPP) indicator set (Fig. 1). The parameter-sparse, process-based agroforestry model Yield-SAFE (van der Werf et al. 2007) is used to compute actual net primary production (NPP) on a plot scale for all three land use scenarios. These plot-scale productivity data are then aggregated to the landscape scale based on datasets from the agent-based model SECLAND (Dullinger et al. 2020), which predicts land use change in the study region by simulating the decision-making process of local agricultural actors. The simple MIAMI (Lieth and Whittaker 1975) model is additionally used to calculate the potential NPP in the study region, an important input parameter to the HANPP framework.

Calculations for the agriculture scenario were performed for the period of 2020–2050. To assess carbon sequestration saturation effects as well as the potential carbon carrying capacity, i.e., the amount of carbon stored in a system with an equally distributed age structure of trees throughout a full harvest cycle, we extended the modeling period for the agroforestry scenarios to the year 2080, corresponding to an assumed harvest cycle of 61 years (de Avila and Albrecht 2017; Martinsson 2001; Pryor 1988).

### Models and input data

Yield-SAFE simulates plot-scale data of aboveground biomass production of trees and crops, enabling quantification of growth dynamics in forestry, arable, and agroforestry systems (van der Werf et al. 2007). Simulations are governed by seven state equations for tree biomass, tree leaf area, number of shoots per tree, crop biomass, crop leaf area index, soil water content, and heat sum, as well as a number of soil-, plant- and site-specific parameters simulating resource acquisition and dry matter accumulation of trees and crops under spatially homogenous competition for light and water (van der Werf et al. 2007; Palma et al. 2016b). The model operates on a daily resolution with inputs of mean temperature, incoming solar radiation, and precipitation. In this study, we used the EcoYield-SAFE web interface (Palma et al. 2016a, b) with standard model calibration for the selected crop and tree species (Palma et al. 2017). Climate data was automatically retrieved from CliPick (Palma 2017), accessing regional climate change datasets for Western Europe from the Coupled Model Intercomparison Project (CMIP5) under the Representative Concentration Pathway RCP4.5 (van Meijgaard et al. 2012; Riahi et al. 2017). To run the model, site-specific inputs of soil, tree, crop, and management parameters were required. A collection of inputs can be found in Table S1.

SECLAND simulates land use decisions taken by agricultural actors in the study region from 2014 to 2050 and outputs geo-referenced raster files depicting each grid cell’s specific land use class in a given year (Dullinger et al. 2020). These actors evaluate their “happiness” on account of workload and generated income to probabilistically decide from a predefined set of ten possible actions (no change, intensification, extensification, direct switch to lowest intensity level, areal expansion, areal reduction, land use change, afforestation, hiring of farm worker, termination). The evaluation process is influenced by framework conditions (yields, market prices, subsidies, preferences) derived from the Shared Socioeconomic Pathways (SSP) (O’Neill et al. 2014, 2017), and model runs were performed for different SSP scenarios. Based on the assumption that the implementation of agroforestry is more likely in a sustainability narrative, we retrieved the dataset from model runs under the sustainability pathway SSP1. This pathway is based upon the increased use of environmentally friendly technologies, global cooperation, a shift towards less resource intensive lifestyles, and a broader acknowledgement of human well-being (van Vuuren et al. 2017). In SECLAND, this narrative was translated into the assumptions of, for example, increased market prices and subsidies for low-input products and energy plants, or a decrease in the maximum of accepted workload (Dullinger et al. 2020).

**Fig. 2:**
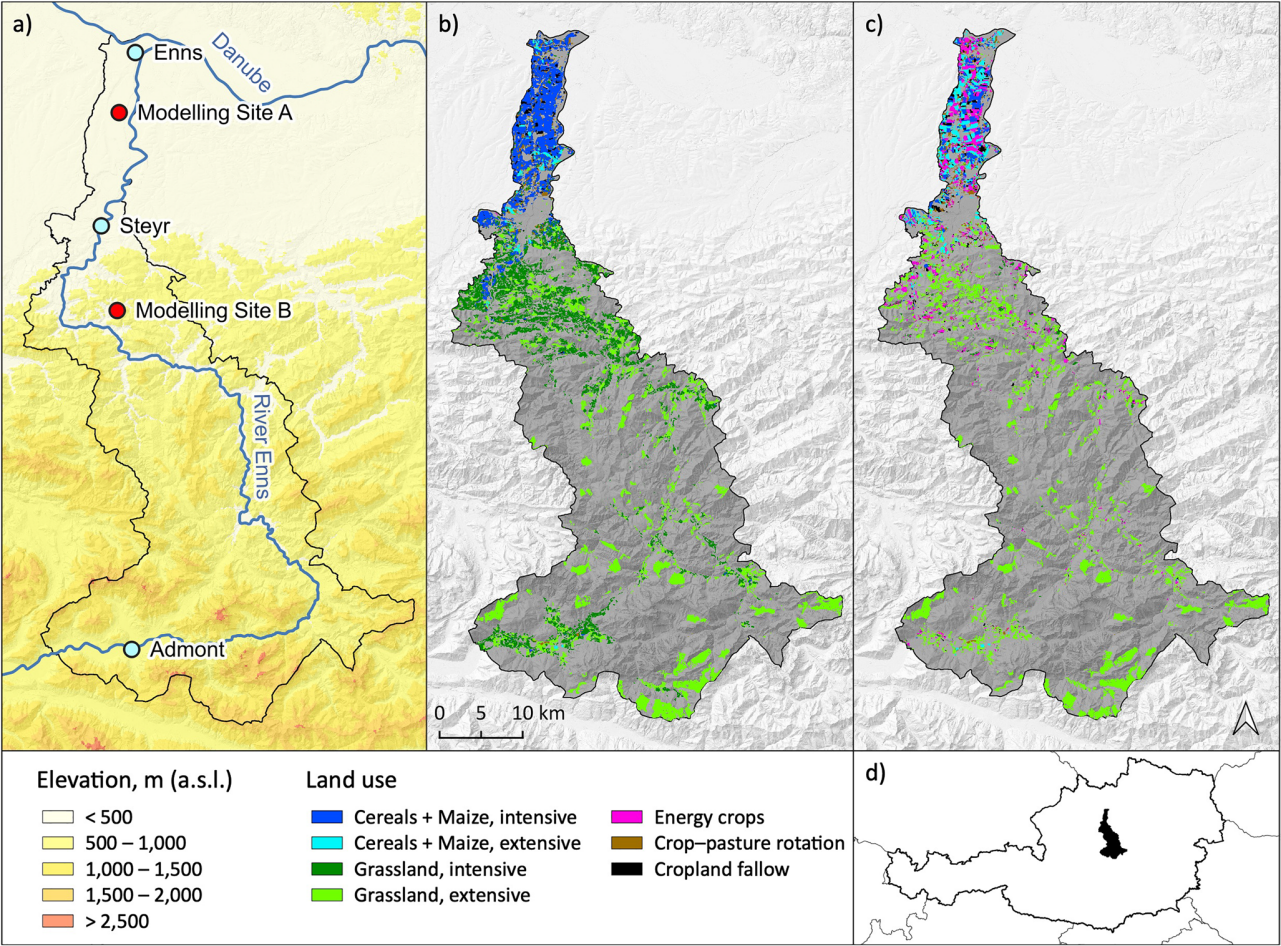
**a** Representation of the study region along the river Enns. **b** Status quo of land use in the base year 2014, when SECLAND model was initialized with data from the Integrated Administration and Control System (IACS). **c** Land use change as simulated by SECLAND model under the Sustainability narrative of the Shared Socioeconomic Pathways (SSP1) for the year 2050. Cropland and grassland agriculture are indicated in color, remaining areas (forests, alpine habitats, infrastructure) are not accounted for in this study. **d** Position of the study region within Austria

Land use change in the study region, as simulated by SECLAND under the SSP1 narrative (Fig. 2 as well as Figure S2 and Table S4), is primarily characterized by a gradual abandonment of grassland (conversion of high-yielding plots to cropland and of low-yielding plots to broad-leaved forest), a trend towards the production of energy crops (conversion from intensive grassland, cereals, and non-cereal crops) and a shift in production intensity towards extensive management (Mayer et al. 2018, 2019). Abandonment and intensification of extensive grassland correspond to the ongoing agro-structural change occurring in many regions of the European Alps (Streifeneder et al. 2007; Lavorel et al. 2017), while the trend towards energy crops and extensive management results from the model’s agents reacting to the SSP1 framework conditions (Dullinger et al. 2020).

The third model we used is the simple MIAMI model (Lieth and Whittaker 1975) to calculate the potential net primary production (NPP) in the study region based on the relationship between annual mean temperature (T, in °C) and annual precipitation (P, in mm) at annual resolution. NPP is assumed to increase with increasing annual average temperature and precipitation, thus always being limited by either. The following formulas apply:

1 $$NPP=\mathit{\min}\left(\ {NPP}_T,{NPP}_P\right)$$

2 $${NPP}_T=3000\times \Big(1+\mathit{\exp}{\left(\mathrm{1,315}-\mathrm{0,119}\times T\right)}^{-1}$$

3 $${NPP}_P=3000\times \Big(1-\mathit{\exp}\left(-0,000664\times P\right)$$

### Scenario development

To assess the effects of agroforestry on biomass harvest and carbon sequestration, we develop three distinct land use scenarios based on land use change simulated by SECLAND from 2020 to 2050. For the extended agroforestry period until 2080, we assume no further land use change beyond the year 2050.

The agriculture scenario (AGR) serves as the baseline, depicting conventional agricultural practice in the region characterized by the strict division of agri- and silviculture (except for small areas exhibiting traditional extensive orchard meadows subsumed in the “extensive grassland” land use class). The agroforestry scenarios (AFS) are counterfactuals exploring the maximum potential effects of agroforestry by assuming implementation on the totality of available agricultural land. Calculations of AFS differentiate between immediate implementation in the year 2020 (AFS-IMM), and gradual implementation in 5-year time-steps between 2020 and 2045 (AFS-GRAD).

We obtain seven aggregated land use classes from the SECLAND datasets (Fig. 2). To allocate the most representative crop species to each land use class, we itemized classes according to the region’s actual prevailing crop cultivars derived from regional agricultural statistics of the federal states of Upper Austria and Styria (STATcube 2019). The “cereals” class, for example, was itemized with wheat, barley, triticale, oats, rye, spelt, and sorghum. Specified crop cultivars were then matched with crop cultivars available for modeling in Yield-SAFE. For each land use class, we selected for further processing the one or two crop cultivars with the highest relevance based on the actual share of agricultural area in the region, as well as the best model fit based on the comparison of a 15-year mean (2000–2014) derived from regional agricultural statistics and model outputs to compensate for singular climate extremes affecting year-to-year productivity. An overview of SECLAND’s land use classes included in the scenarios, corresponding representative species identified from agricultural statistics, as well as the species equivalents selected from Yield-SAFE is compiled in Table S2.

In AFS we assume prototypical silvoarable alley cropping plots of 100 × 100 m. Regardless of the crop cultivar, every plot includes 4 tree rows of wild cherry (*Prunus avium L.*) with 20 trees each, resulting in 80 trees ha^-1^. This configuration corresponds to agroforestry design adequate to the study region (Reeg et al. 2009; Kaeser et al. 2011; Sereke et al. 2015; Crous-Duran et al. 2018). We selected wild cherry (*Prunus avium L.*) as tree species from Yield-SAFE because (i) it occurs naturally throughout European temperate forests and is found at colline to submontane altitudes up to an elevation of 1700 m a.s.l. in the Northern and Central Alps (Ducci et al. 2013; Welk et al. 2016); (ii) it provides fruit as a potential food source and high value timber for long-lived veneer products; and (iii) it is beneficial to biodiversity as an integral part of sustainable land use (Schmidt 2010; Welk et al. 2016) and has a high aesthetic, cultural, and touristic value, fitting the landscape’s historic characteristics (Herzog 1998; Styrian Eisenwurzen Nature & Geopark 2020). The assumed harvest cycle of 61 years is based on the fact that wild cherry features stagnant growth rates and increased susceptibility to pests and disease after 50 years, making a rotation period of 50 to maximum 90 years recommendable (de Avila and Albrecht 2017; Martinsson 2001; Pryor 1988).

### Human Appropriation of Net Primary Production

To quantify carbon dynamics and trade-offs between carbon sequestration and biomass provision, we build upon the Human Appropriation of Net Primary Production (HANPP) framework. HANPP expresses the amount of carbon appropriated by humans in a given year through harvest and land conversion (Haberl et al. 2014). It serves as a pressure indicator for ecosystems by denoting the amount of energy withdrawn from the trophic levels of the food chain (Erb et al. 2016). Here, we restricted calculations to aboveground NPP (denoted by the prefix “a”) on crop- and grassland, while belowground NPP as well as forest land, infrastructure areas, and other land are excluded.

**Fig. 3:**
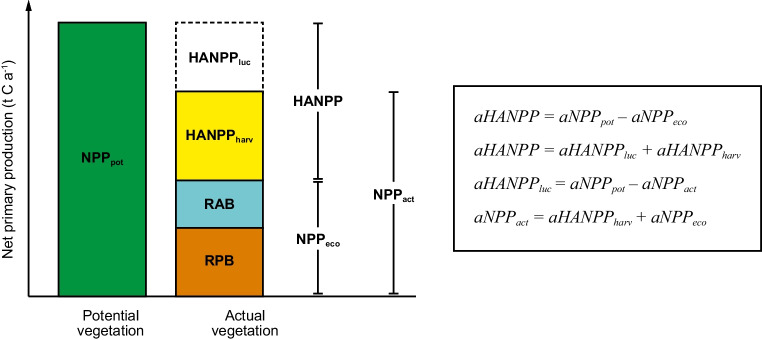
Fluxes of net primary production (NPP) contributing to the indicator framework Human Appropriation of Net Primary Production (HANPP) and corresponding equations. Adapted from Haberl et al. (2014) and Erb et al. (2009)

The HANPP framework is composed of a range of individual indicators (Table 1), including potential and actual net primary production (aNPP_pot_ and aNPP_act_); effects of land conversion (aHANPP_luc_); biomass harvested, grazed, or destroyed during harvest (aHANPP_harv_); as well as biomass remaining in the ecosystem after harvest (aNPP_eco_). Yields, used residues, and unused residues are expressed individually as parts of aHANPP_harv_ and depict final crop, grass, and cherry yields, harvest residues used for economic activities (e.g. straw used as animal feed or litter) as well as harvest residues left on the field (e.g. stubble or roots that die off during harvest). To explicitly account for carbon sequestration, we extend the framework so that aNPP_eco_ is further decomposed into remaining annual biomass (RAB) and remaining perennial biomass (RPB), building on methodology applied in Niedertscheider et al. (2017) and Guzmán et al. (2018). While RAB denotes vegetation that remains in the ecosystem but dies off every year (like annual weeds on fallow land or leaf litter of trees), RPB accumulates during tree growth to build up vegetation carbon stocks in woody biomass. The coinciding trends in aHANPP_harv_ and RPB thus inform about trade-offs between biomass provision and carbon sequestration, while trends in aNPP_act_ inform about changes in total agroecological productivity. Central relations between these components and corresponding equations are depicted in Fig. 3. All indicators refer to annual fluxes of carbon and are given in t C yr^-1^ or percent of aNPP_pot_.

**Table 1:** Overview of the components included in the Human Appropriation of Net Primary Production indicator framework

HANPP indicator	Description
NPP_pot_	Potential NPP that would prevail without human intervention
NPP_act_	Actual NPP under the prevailing land use
HANPP_luc_	NPP lost (or gained) through land use change
HANPP_harv_	NPP harvested, grazed, or destroyed during harvest
Yields	Final crop, grass, and cherry yields
Used residues	Harvest residues used for economic activities
Unused residues	Harvest residues left on the field
NPP_eco_	NPP remaining in the ecosystem after harvest
RAB	Remaining annual biomass that dies off every year
RPB	Remaining perennial biomass that accumulates to build up carbon stocks

In contrast to other HANPP studies, usually deriving NPP_act_ from agricultural and forest statistics and remotely sensed data (e.g., Mahbub et al. 2019; Fetzel et al. 2016; Gingrich et al. 2015; Haberl et al. 2014), here we used Yield-SAFE to simulate aNPP_act_ and MIAMI to calculate aNPP_pot_. Both models provide production data in dry matter (DM), except for cherries, which are reported in fresh weight. An overview of calculations, expansion factors, and coefficients to calculate aNPP_pot_, aNPP_act_, aNPP_eco_, and aHANPP_harv_ components from MIAMI and Yield-SAFE outputs can be found in Table S3.

## Results

**Fig. 4:**
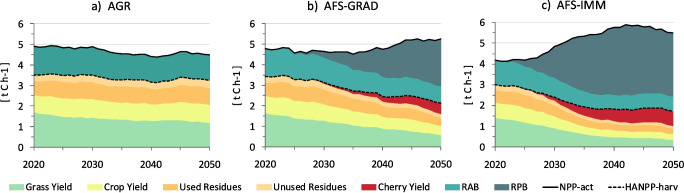
Composition of aNPP_act_ in **a** the agriculture scenario (AGR), **b** the gradual agroforestry scenario (AFS-GRAD), and **c** the immediate agroforestry scenario (AFS-IMM) between 2020 and 2050 in area-weighted average t C ha^-1^ yr^-1^. The sum of crop and grass yields as well as used and unused residues equals biomass harvest (aHANPP_harv_); the sum of remaining annual biomass (RAB) and remaining perennial biomass (RPB) equals biomass in the ecosystem after harvest (aNPP_eco_); the sum of aHANPP_harv_ and aNPP_eco_ equals the actual net primary production (aNPP_act_)

**Fig. 5:**
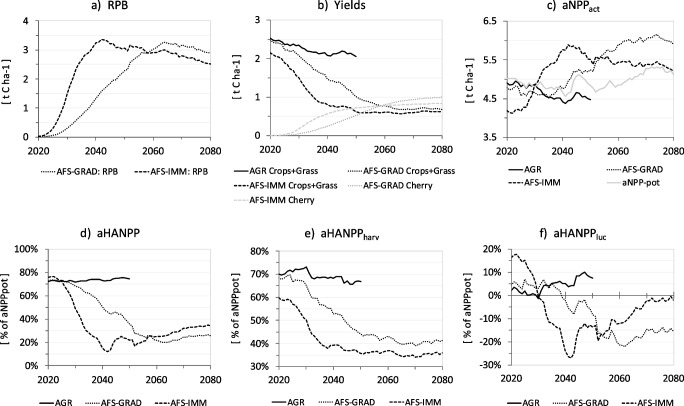
Comparison of the three modeling scenarios for the extended period of 2020 and 2080. Graphs show **a** remaining perennial biomass (RPB), **b** yields of crops + grass (combined) and cherry, **c** actual and potential net primary production (aNPP_act_, aNPP_pot_), **d** total aHANPP, **e** biomass harvest (aHANPP_harv_), and **f** anthropogenic land use change (aHANPP_luc_). Values are given in **a**–**c** area-weighted average t C ha^-1^ yr^-1^ and **d**–**f** percent of aNPP_pot_.

The hypothetical transition to agroforestry in the study region profoundly alters the carbon dynamics of the agroecosystem (Figs. 4 and 5, and Table S5). While aNPP_pot_ remains relatively constant (just under 5 t C ha^-1^ yr^-1^ between 2020 and 2050), aNPP_act_ declines from 4.8 to 4.5 t C ha^-1^ yr^-1^ and aHANPP_harv_ from 2.5 to 2.1 t C ha^-1^ yr^-1^ in AGR. These declines result from land use change simulated by SECLAND. This development leads from a neutral to a slightly positive aHANPP_luc_ of 0.4 t C ha^-1^ yr^-1^ (corresponding to 8% of aNPP_pot_) in 2050. While both AFS result in land use extensification (i.e., a reduction of aHANPP_harv_), aNPP_act_ nevertheless increases during the same timeframe, from 4.8 to 5.2 t C ha^-1^ yr^-1^ in AFS-GRAD and 4.2 to 5.5 t C ha^-1^ yr^-1^ in AFS-IMM (Fig. 4b and c). These changes in aNPP_act_ are driven by the co-existence of trees and crops. Tree growth thereby shapes the curve of RPB by retaining a large fraction of biomass in the ecosystem. According to the simulations, wild cherries feature a strong growth rate during the first 20 years, after which they sustain a considerably high but slightly decreasing annual increment until 2080 (Fig. 5a). In AFS-IMM, RPB peaks in 2042 with an annual increment of 3.4 t C ha^-1^ yr^-1^ and remains above 2.5 t C ha^-1^ yr^-1^ thereafter. AFS-GRAD shows a delayed and less pronounced development with annual tree growth increasing steadily but at a slower pace, reaching 2.3 t C ha^-1^ yr^-1^ in 2050 and an annual maximum increment of 3.3 t C ha^-1^ yr^-1^ in 2064.

Resource competition in the AFS negatively affects crop production. Crop and grass yields decline substantially between 2020 and 2050, from 2.5 to 1 t C ha^-1^ yr^-1^ (− 60%) in AFS-GRAD and 2.1 to 0.6 t C ha^-1^ yr^-1^ (− 71%) in AFS-IMM (Fig. 4). Compared to crop and grass yields in AGR in 2050 (2.1 t C ha^-1^ yr^-1^), this corresponds to a deficit of − 52% in AFS-GRAD and − 71% in AFS-IMM. Decreases of aHANPP_harv_, however, are substantially attenuated, as cherry fruit production significantly sets off crop and grass yield losses, starting as early as 2030 in AFS-IMM. By 2050, cherry yields in AFS-IMM increase up to the point at which the yearly amount of harvested fruit (0.7 t C ha^-1^ yr^-1^) is larger than that of harvested crops and grass (Fig. 5b). In AFS-GRAD, cherry yields increase more slowly but also become a significant factor by 2050. Compared to AGR in 2050, total aHANPP_harv_ declines by − 34% in AFS-GRAD and − 47% and AFS-IMM (Fig. 5e). Looking at the extended modeling period until 2080, crop and grass yields in AFS-IMM and AFS-GRAD do not decrease any further beyond 2050 and 2065, respectively, and cherry yields likewise stabilize between 0.8 and 1 t C ha^-1^ yr^-1^ (Fig. 5b). From this we infer that no further significant increase in the interception of solar radiation and competition for water occurs after 20–30 years in tree age. This is, nevertheless, opposed by sustained RPB in both AFS until 2080.

From a productivity perspective, tree growth and cherry production in both AFS overcompensate for losses in crop and grass yields. This effect is expressed in significantly higher aNPP_act_ and negative aHANPP_luc_ values (Fig. 5c and f). Actual and potential aNPP, nevertheless, converge again in AFS-IMM, where trees are of uniform age. In AFS-GRAD, the broader distribution of tree age leads to a moderated development of aNPP_act_ and a more constant rate of negative aHANPP_luc_ over time. If taken together, effects on biomass production, harvest, and the build-up of carbon stocks in AFS-IMM and AFS-GRAD result in a drastic reduction of aHANPP from > 70 to < 35% of aNPP_pot_ from 2020 to 2080 (Fig. 5d). This development indicates a strong reduction of anthropogenic pressure on the agroecosystem in the study region while system-level productivity (aNPP_act_) is increased, despite the extensification trend and declining yields. In AGR, on the contrary, aHANPP rises slightly from 72% to 74% of aNPP_pot_ (2020–2050) as a direct result of land use change simulated by SECLAND.

**Fig. 6:**
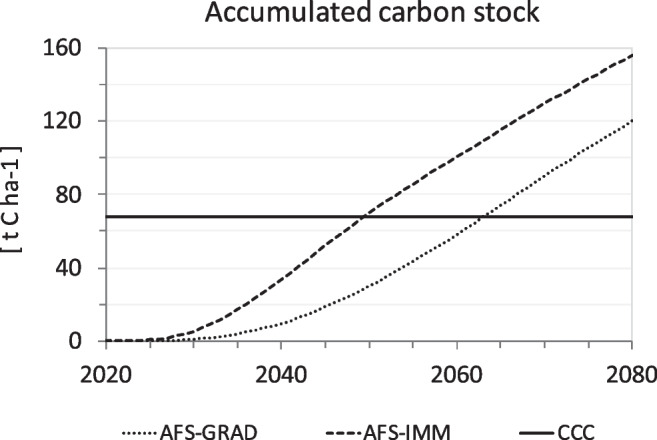
Development of the accumulated carbon stock in AFS-GRAD and AFS-IMM from 2020 to 2080 as well as the carbon carrying capacity (CCC) in the study region, in t C ha^-1^. Intersections with the solid line depict the year in which the region’s carbon carrying capacity is reached in the respective scenario

Accumulated carbon stocks reach 120 and 156 t C ha^-1^ (2.6 and 3.37 Mt C in the study region) between 2020 and 2080 in AFS-GRAD and AFS-IMM, respectively (Fig. 6). A significant saturation in tree productivity was not simulated within the extended study period of 61 years (as trees accumulate >2.5 t C ha^-1^ yr^-1^ until 2080). While increases in tree height and diameter at breast height stagnate significantly from 2050 onwards, strong growth of branch wood continues unabatedly, consistent with development of tree structures associated with cherry fruit production (Sheppard and Spiecker 2015). Extending the modeling period to the year 2080 also enabled the calculation of the carbon carrying capacity, i.e., the amount of carbon potentially being stored permanently in the agroforestry system if tree age structure were equally distributed throughout the assumed harvest cycle of 61 years. Carbon carrying capacity thus corresponds to the net carbon sink in AFS. Carbon carrying capacity reaches 67.5 t C ha^-1^ at a mean tree age of 30.5 years, corresponding to roughly two-thirds of the actual carbon stock per hectare in an Austrian mixed forest (Erb 2004). This is a substantial amount which leads to a total potential long-term carbon pool of 1.46 Mt C in the study area. Carbon carrying capacity is reached in 2063 in AFS-GRAD and 2049–2050 in AFS-IMM (Fig. 6).

## Discussion

Evaluation of ES trade-offs is a central area of research to inform sustainable land use policy (Crossman et al. 2013; Schirpke et al. 2013; Bennett et al. 2009; Rodríguez et al. 2006) and mountain agriculture (Locatelli et al. 2017; Flury et al. 2013; Briner et al. 2013a). To our knowledge, there exist few studies (e.g., Kay et al. 2018) that quantify potential impacts of agroforestry implementation on biomass harvest and carbon sequestration on a landscape scale in a temperate European context. To close this research gap, we integrated data from a process-based agroforestry model and an agent-based land use model and calculated carbon dynamics in an alpine agroecosystem for three counterfactual land use scenarios.

Exploring biophysical effects at the landscape scale demands systematic trade-offs between accuracy and generalizability, pertaining to methodological complexity, data requirements, and robustness. Applied models and data vary in temporal and spatial resolution, ranging from daily (Yield-SAFE) to yearly (SECLAND, MIAMI) time steps as well as from plot to federal state levels (modeled and statistical data). Additionally, MIAMI model does not account for differences in NPP resulting from seasonal variation in climate (Zaks et al. 2007; Field et al. 1995), producing imprecisions on smaller timescales not affecting our overall results. The use of pre-existing Yield-SAFE calibrations led to relatively large deviations of simulated and statistically reported yields of some cultivars (at the level of federal provinces), which were subsequently not included in the study, reducing the number of cultivars to those for which the pre-existing calibration produced the most plausible results (Figure S1). While yield simulations could thus not be fully accurate, evaluation of trade-offs was nevertheless consistent, as all three scenarios relied on the same modeling procedures and accounting approach.

By analyzing biomass provision and carbon sequestration, we focus on two ES that are most affected by aboveground biomass. Although some root biomass is destroyed during harvest, the vast majority of biomass extraction takes place aboveground (Krausmann et al. 2013). Carbon sequestration is also dominated by aboveground biomass when tree cover expands (Le Noë et al. 2021; Gingrich et al. 2007), but soil organic carbon represents a large additional carbon pool that we excluded from analysis. While it is not straight-forward to quantify, literature on temperate agroforestry systems suggests that soil organic carbon may create significant additional carbon sinks under agroforestry due to the input of tree leaf litter and root-derived carbon inputs (Lim et al. 2018; Cardinael et al. 2017; Pardon et al. 2017; Lorenz and Lal 2014). Therefore, probably, our study underestimates the carbon sequestration potential of agroforestry, while more accurately representing its biomass provision.

Agroforestry systems allow for complementary use of resources (i.e., solar radiation, water and nutrients) through the production of different products or mixture of different species due to ecological niche differentiation (Cannell et al. 1996; Smith et al. 2013). This can result in a higher land equivalent ratio, i.e., a yield advantage of intercropping as compared to sole cropping (Mead and Willey 1980), which has been shown in various agroforestry studies (e.g., Lehmann et al. 2020; Seserman et al. 2018; Sharrow and Ismail 2004). Many studies, however, are characterized by varying system designs and species compositions, management practices, climatic and soil conditions, spatial and temporal scales, as well as research methodology, making it difficult to arrive at consistent comparisons (Torralba et al. 2016; Rivest et al. 2013). Additionally, there still exist relatively little consistent experimental field data from systems with mature tree components (Brown et al. 2018; Miller et al. 2020).

Torralba et al. (2016), for example, showed that positive effects on yields are more significant in Mediterranean and Pannonian than in Alpine and Continental biogeographical regions. Primarily negative yield effects have been documented in a variety of relevant studies, with a range of results including − 29% in mean annual biomass yields in four Swiss agroforestry landscape test sites (Kay et al. 2018); − 8% (winter wheat) to − 65% (forage maize) in yields with 48-year-old trees (*Prunus avium, Populus* sp.*, Juglans regia*, and *Sorbus torminalis*) in Belgium (Pardon et al. 2018); − 51% (spring wheat, potatoes and squash with willow) in the UK to + 16% (winter wheat with willow, alder, and hazelnut) in Denmark (Lehmann et al. 2020); and neutral effects (winter wheat and winter oilseed with 5–6-m high poplar clones) in systems with wide cropping alleys (of 48 and 96 m, contrasting 25 m in this study) in Germany (Swieter et al. 2019). Yield reductions estimated in our study of − 52% in AFS-GRAD and − 71% in AFS-IMM, as compared to AGR in 2050, are still within scope but more severe than the majority of reported values. This might equally be due to an over-estimation of resource competition in Yield-SAFE, or to the effect of the regional agroforestry specificities.

A recent compilation (Lawson et al. 2019) reports carbon sequestration rates in temperate climates ranging from 0.1 to 13 ha^-1^ yr^-1^. One relevant study, including four silvoarable and silvopastoral systems (*Prunus avium and Juglans regia x nigra*) aged 18–41 years in France, reports aboveground carbon sequestration rates of 0.5–1.5 t C ha^-1^ yr^-1^ (Cardinael et al. 2017). The IPCC default coefficient for aboveground woody biomass in cropping systems containing perennial species in temperate climates is 2.1 t C ha^-1^ yr^-1^ (63 t C ha^-1^ over 30 years) (IPCC 2006). And Dixon (1995) calculated a global median aboveground carbon storage in the vegetation of agroforestry systems of 1.4 t C ha^-1^ yr^-1^ (70 t C ha^-1^ over the period of 50 years). By comparison, the average accumulation rate in this study was computed at 0.9 and 2.1 t C ha^-1^ yr^-1^ for the period of 2020–2050 as well as 1.9 and 2.4 t C ha^-1^ yr^-1^ for the period of 2020–2080 in AFS-GRAD and AFS-IMM, respectively. These results correspond to the lower end of the range compiled by Lawson et al. (2019) and coincide well with the latter estimates.

While our study quantifies the maximum impacts by including all available agricultural land in the region, results suggest that such a radical agroforestry implementation is not conducive to socio-ecological sustainability. To limit socio-economic impacts of estimated yield reductions, agroforestry implementation appears to be better suited to marginal instead of high-yielding lands. In particular, extensively managed meadows and pastures are an attractive opportunity in the study region, given their relatively low productivity and large area extent (STATcube 2019) as well as the historical connection to agroforestry in the region (Styrian Eisenwurzen Nature & Geopark 2020; Buttler et al. 2008; Herzog 1998). These characteristics are similar in many alpine and sub-alpine grasslands, which are increasingly abandoned and compromised by succession (Schirpke et al. 2017; Streifeneder et al. 2007; Tasser et al. 2005).

Assuming the implementation of agroforestry systems on Austria’s extensively managed grasslands, amounting to roughly 0.64 Mha in 2018 (BMNT 2019)^1^ and using data from AFS-GRAD with a carbon carrying capacity of 67.5 t C ha^-1^ in the year 2063, the net carbon sink would amount to roughly 43.4 Mt C or 0.99 Mt C yr^-1^ between 2020 and 2063. This corresponds to c. 4.5% of annual GHG emissions (21.75 Mt C in 2016), c. 84.3% of the annual net carbon sink from forest land (1.17 Mt C in 2016)^2^ or c. 49.5% of the annual emissions from agriculture (1.99 Mt C in 2016) in Austria (Anderl et al. 2018). Furthermore, compared to the actual carbon stocks in mixed forests in Austria, estimated at 96 t C ha^-1^ by Erb (2004), the carbon carrying capacity of 67.5 t C ha^-1^ amounts to just over two-thirds of that value, representing a substantial amount of stored carbon while the agroecosystem continuously provides biomass products. From these comparisons, we conclude that the potential aboveground carbon sequestration rate of the silvoarable agroforestry system studied here could substantially contribute to climate change mitigation by (i) significantly offsetting GHG emissions for almost three decades after agroforestry implementation and (ii) creating a permanent net carbon pool that additionally provides a range of provisioning and regulating ES.

As such, agroforestry can contribute to interlink different aspects of regional development, land use, and climate policy. Additional synergies arise, in this case, by counteracting biodiversity loss caused by grassland abandonment as well as by increasing cultural ES, adding aesthetic and touristic value to the landscape. Potential synergies between climate change mitigation and adaptation also result in increased risk abatement (e.g., by reducing susceptibility to extreme weather events) and resilience by diversifying sources of income (Matocha et al. 2012; Hernández-Morcillo et al. 2018). This provides further incentives to establish agroforestry as part of sustainable land use and climate policy.

## Conclusion

Here, we quantify trade-offs between carbon sequestration and biomass provision inherent to a hypothetical transition to agroforestry on a landscape scale in the alpine long-term socio-ecological research region *Eisenwurzen* in Austria. The study links to the discourse about sustainable land use policy by providing a reference frame for the assessment of agroforestry impacts on the carbon dynamics in the land agroecosystem. Our estimations indicate increased NPP and high carbon sequestration potential, in parallel with strong reductions in yields.

We conclude that agroforestry qualifies as an option for developing sustainable mountain agriculture, but increasing monetary remuneration of carbon sequestration and other regulating ES through adequate land use policy is needed to enable agroforestry implementation, where appropriate. To this end, future research should focus on quantifying trade-offs and synergies on a landscape scale involving different agroforestry compositions on a gradient of pre-existing land use intensity as well as additional land use types (e.g., forest), managing a balance of provisioning and regulating ES within a region’s larger socio-ecological context.

## Supplementary Information

ESM 1 (DOCX 109 kb)

## Footnotes

1 This is a crude estimation that does not take into account any bio-physical, socio-economic, or legal restrictions that might apply in reality, likely resulting in an overestimation of the technical carbon sequestration potential.

2 This number relates to the net carbon removals on forest land, without consideration of removals from harvested wood products as well as land-use change emissions.
