# Supplementary material for: Agroforestry trade-offs between biomass provision and aboveground carbon sequestration in the alpine Eisenwurzen region, Austria
Source: Reg Environ Change. 2021 Jul 21;21(3):77. doi: 10.1007/s10113-021-01794-y (PMC8550091; doi:10.1007/s10113-021-01794-y)
Supplement: Supplementary file 1 — (DOCX 109 kb) [file 10113_2021_1794_MOESM1_ESM.docx]

**Supplementary Information (SI)**

***Agroforestry trade-offs between biomass provision and carbon sequestration in the alpine LTSER region Eisenwurzen, Austria***

Bastian Bertsch^1*^, Claudine Egger^1^, Veronika Gaube^1^, Simone Gingrich^1^

*^1^ Institute of Social Ecology (SEC), Department of Economics and Social Sciences (WiSo), University of Natural Resources & Life Sciences, Vienna (BOKU), Schottenfeldgasse 29, 1070 Vienna, Austria*

** Corresponding author:* [*bastian.bertsch-hoermann@boku.ac.at*](mailto:bastian.bertsch-hoermann@boku.ac.at)

**Yield-SAFE model inputs**

Table S1: Selection of EcoYield-SAFE input parameters specified for the model runs in this study. Location and soil parameters vary for the two modelling sites, indicated in parenthesis.

| EcoYield-SAFE Parameters | Value |
| --- | --- |
| Water limitation | Yes |
| Latitude (Cropland) | 48,160073 |
| Longitude (Cropland) | 14,451603 |
| Latitude (Grassland) | 47,945322 |
| Longitude (Grassland) | 14,443828 |
| Soil ID (Cropland) | Medium-Fine |
| Soil ID (Grassland) | Medium |
| Soil depth (mm) | 700 |
| Plant density (trees ha⁻¹) | 80 |
| DOY to plant | 60 |
| Alley width | 2 |
| Fruit production | Yes |
| Canopy effect on evapotranspiration | On |
| Tree height for temperature effect on evapotranspiration | 4 |
| Maximum difference in temperature in summer | 5 |
| Maximum difference in temperature in winter | 2 |
| Tree wind effect on evapotranspiration | On |
| Tree height for wind effect on evapotranspiration | 1 |

**Scenario development**

Table S2: Compilation of SECLAND’s land-use classes included in the scenarios, corresponding representative species identified from agricultural statistics as well as the species-equivalents selected from Yield-SAFE.

| SECLAND land-use class | Agriculture statistics | Yield-SAFE cultivar |
| --- | --- | --- |
| Energy Crop | Winter oilseed | Oilseed |
| Non-cereal crop | Grain maize incl. CCM | Grain maize |
| Cereal | Winter barley | Barley |
| Cereal | Winter wheat | Winter wheat |
| Misc. arable land | Egart | Grass (extensive CH) |
| Fallow land | n.d. | Grass (extensive CH) |
| Extensive pasture / meadow | Meadow, one and two mowings | Grass (extensive CH) |
| Intensive pasture / meadow | Meadow, three and more mowings | Grass (80% Dactylus) |

**HANPP coefficients and expansion factors**

Dry matter was converted into carbon by assuming 50% carbon content (CC) (IPCC 2006). Water content (WC) for cherries was assumed at 83% (Walker et al. 2011; Wojdyło et al. 2014). To account for productivity losses caused by extensive management practices, low-input factors (LI) were derived from yield gaps between organic and conventional farming in Austria (Resl and Brückler 2016). aHANPP_harv_ components were calculated from aNPP_act_ by subtracting pre-harvest losses (PHL) caused through herbivory and weeds. Corresponding factors for crops and grass (Krausmann 2001; Haberl et al. 2007, SI), cherries (Öztürk et al. 2010) as well as harvest indices (HI) and recovery rates (RR) to calculate final yields and residues (Wirsenius 2000, 2003) were applied. Harvest on grassland was calculated by applying a factor of 0.75 to aNPP_act_, representing the maximum fraction assumed to be harvestable through grazing or mowing (Haberl et al. 2007, SI).

Table S3: Coefficients to calculate aNPP_act_ and aHANPP_harv_ components from Yield-SAFE model outputs. CC: carbon content; WC: water content; LI: low-input factor; PHL: pre-harvest loss factor; HI: harvest index; RR: recovery rate.

| **Cultivars** | **CC** | **WC** | **LI** | **PHL** | **HI** | **RR** |
| --- | --- | --- | --- | --- | --- | --- |
| Cherry fruit | 0.5 | 0.17 | - | 1.09 | - | - |
| Winter Wheat | 0.5 | - | 0.6 | 1.14 | 0.5 | 0.7 |
| Barley | 0.5 | - | 0.6 | 1.14 | 0.45 | 0.7 |
| Grain Maize | 0.5 | - | 0.65 | 1.14 | 0.45 | 0.7 |
| Oilseed | 0.5 | - | 0.65 | 1.14 | 0.35 | 0.7 |
| Grass (ext. CH) | 0.5 | - | - | 1.14 | 0.75 | - |
| Grass (80% Dactylus) | 0.5 | - | - | 1.14 | 0.75 | - |
| Wild cherry | 0.5 | - | - | - | - | - |

aNPP_act_ of trees was calculated as the difference between the minimum and maximum stand biomass per year (expressed as *Bt_tonha* in Yield-SAFE). Leaf fall was calculated as the difference between the largest and second largest annual value, as biomass values decline every year from October to November, representing fallen leaves. Annual RPB was calculated as aNPP_act_ of trees minus leaf fall.

Data from MIAMI model contains estimates for below-ground NPP of 17% (Haberl 1995) which was subtracted to determine aNPP_pot_.

All values were calculated as 5-year averages to reduce the impact of stochastic weather events (Haberl et al. 2007; Krausmann et al. 2008).

**SECLAND land-use change**

Table S4: Relevant land use classes and their respective area extent in hectares, 2020 and 2050, as well as the change in area in percent.

| Land use class | 2020 | 2050 | Change in % |
| --- | --- | --- | --- |
| Energy crop | 724.5 | 2,208.4 | 205% |
| Energy crop low-input | 115.1 | 824.9 | 617% |
| Non-cereal crop | 2,180.8 | 294.4 | -87% |
| Non-cereal low-input | 1,836.5 | 1,917.1 | 4% |
| Cereal | 1,528.7 | 628.8 | -59% |
| Cereal low-input | 996.4 | 928.4 | -7% |
| Misc. arable land | 595.5 | 589.5 | -1% |
| Fallow land | 2,070.1 | 2,166.4 | 5% |
| Extensive pasture | 10,149.6 | 8,271.9 | -19% |
| Extensive meadow | 4,534.9 | 3,341.9 | -26% |
| Intensive pasture | 1,732.7 | 196.2 | -89% |
| Intensive meadow | 1,879.8 | 269.7 | -86% |

Figure S1: Development of agricultural land use classes in thousand hectares, 2020–2050.


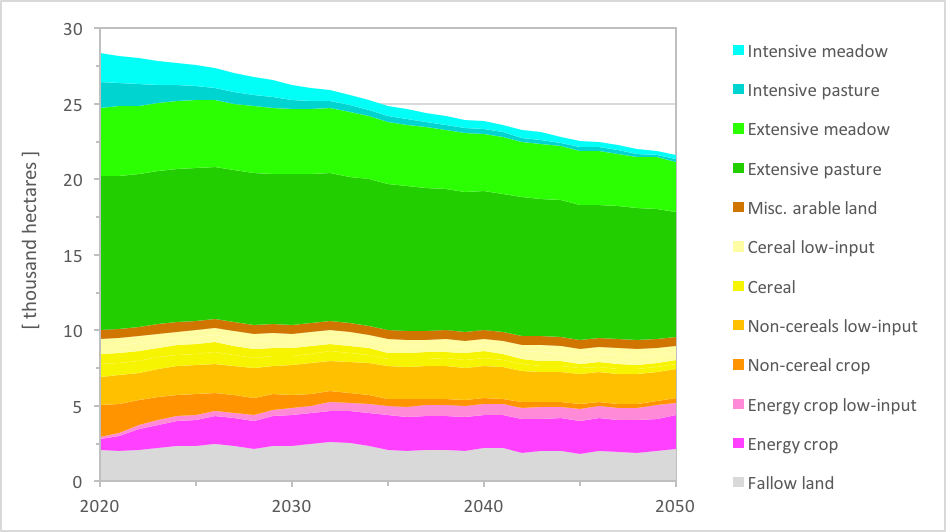


**aHANPP mean values**

Table S5: Comparison of aHANPP indicators for the periods of 2020–2050 and 2020–2080. Values represent the mean of area-weighted average carbon flows and stocks in t C ha^-1^.

|  |  | 2020–2050 | | |  | 2020–2080 | |
| --- | --- | --- | --- | --- | --- | --- | --- |
| HANPP indicator |  | AGR | AFS-IMM | AFS-GRAD |  | AFS-IMM | AFS-GRAD |
| aNPP_pot_ |  | 4.87 | 4.87 | 4.87 |  | 4.95 | 4.95 |
| aNPP_act_ |  | 4.66 | 5.11 | 4.82 |  | 5.26 | 5.34 |
| aNPP_eco_ |  | 1.29 | 2.91 | 1.99 |  | 3.26 | 2.88 |
| RPB |  | - | 2.05 | 0.91 |  | 2.45 | 1.93 |
| RAB |  | 1.29 | 0.85 | 1.08 |  | 0.81 | 0.95 |
| aHANPP |  | 3.57 | 1.96 | 2.88 |  | 1.69 | 2.06 |
| aHANPP_luc_ |  | 0.21 | -0.25 | 0.05 |  | -0.31 | -0.40 |
| aHANPP_harv_ |  | 3.36 | 2.20 | 2.83 |  | 2.00 | 2.46 |
| Yield, Crops |  | 0.87 | 0.47 | 0.70 |  | 0.37 | 0.53 |
| Yield, Grass |  | 1.37 | 0.76 | 1.09 |  | 0.55 | 0.75 |
| Yield, Fruit |  | - | 0.37 | 0.18 |  | 0.58 | 0.51 |
| Used residues |  | 0.79 | 0.43 | 0.61 |  | 0.35 | 0.48 |
| Unused residues |  | 0.34 | 0.18 | 0.26 |  | 0.15 | 0.20 |

**References**

Haberl H (1995) Menschliche Eingriffe in den natürlichen Energiefluß von Ökosystemen. Sozio-ökonomische Aneignung von Nettoprimärproduktion in den Bezirken Österreichs. Universität Wien

Haberl H, Erb KH, Krausmann F, et al (2007) Quantifying and mapping the human appropriation of net primary production in earth’s terrestrial ecosystems. Proceedings of the National Academy of Sciences 104:12942–12947. https://doi.org/10.1073/pnas.0704243104

IPCC (2006) Guidelines for National Greenhouse Gas Inventories. IPCC

Krausmann F (2001) Land use and industrial modernization: an empirical analysis of human influence on the functioning of ecosystems in Austria 1830–1995. Land Use Policy 10

Krausmann F, Erb K-H, Gingrich S, et al (2008) Global patterns of socioeconomic biomass flows in the year 2000: A comprehensive assessment of supply, consumption and constraints. Ecological Economics 65:471–487. https://doi.org/10.1016/j.ecolecon.2007.07.012

Öztürk FP, Kaçal E, Sarısu HC, et al (2010) ECONOMIC EVALUATION OF PREHARVEST AND HARVEST LOSSES IN “0900 ZIRAAT” SWEET CHERRY CULTIVAR. Acta Horticulturae 261–267. https://doi.org/10.17660/ActaHortic.2010.877.29

Resl T, Brückler M (2016) Erträge des österreichischen Biolandbaus im Vergleich zu konventioneller Produktion

Walker RP, Battistelli A, Moscatello S, et al (2011) Phosphoenolpyruvate carboxykinase in cherry (Prunus avium L.) fruit during development. Journal of Experimental Botany 62:5357–5365. https://doi.org/10.1093/jxb/err189

Wirsenius S (2003) Efficiencies and biomass appropriation of food commodities on global and regional levels. Agricultural Systems 77:219–255. https://doi.org/10.1016/S0308-521X(02)00188-9

Wirsenius S (2000) Human Use of Land and Organic Materials: Modeling the Turnover of Biomass in the Global Food System. Chalmers University of Technology, Göteborg University

Wojdyło A, Nowicka P, Laskowski P, Oszmiański J (2014) Evaluation of Sour Cherry ( Prunus cerasus L.) Fruits for Their Polyphenol Content, Antioxidant Properties, and Nutritional Components. Journal of Agricultural and Food Chemistry 62:12332–12345. https://doi.org/10.1021/jf504023z
